# The Road to Better Management in Resistant Hypertension—Diagnostic and Therapeutic Insights

**DOI:** 10.3390/pharmaceutics13050714

**Published:** 2021-05-13

**Authors:** Elisabeta Bădilă, Cristina Japie, Emma Weiss, Ana-Maria Balahura, Daniela Bartoș, Alexandru Scafa Udriște

**Affiliations:** 1 Faculty of Medicine, “Carol Davila” University of Medicine and Pharmacy Bucharest, 050474 Bucharest, Romania; elisabeta.badila@umfcd.ro (E.B.); emma.weiss@umfcd.ro (E.W.); ana-maria.balahura@umfcd.ro (A.-M.B.); daniela.bartos@umfcd.ro (D.B.); alexandru.scafa@umfcd.ro (A.S.U.); 2 Department of Internal Medicine, Clinical Emergency Hospital of Bucharest, 014461 Bucharest, Romania; 3 Department of Cardiology, Clinical Emergency Hospital of Bucharest, 014461 Bucharest, Romania

**Keywords:** resistant hypertension, pseudoresistance, adherence, diuretic, mineralocorticoid receptor antagonist, lifestyle measure

## Abstract

Resistant hypertension (R-HTN) implies a higher mortality and morbidity compared to non-R-HTN due to increased cardiovascular risk and associated adverse outcomes—greater risk of developing chronic kidney disease, heart failure, stroke and myocardial infarction. R-HTN is considered when failing to lower blood pressure below 140/90 mmHg despite adequate lifestyle measures and optimal treatment with at least three medications, including a diuretic, and usually a blocker of the renin-angiotensin system and a calcium channel blocker, at maximally tolerated doses. Hereby, we discuss the diagnostic and therapeutic approach to a better management of R-HTN. Excluding pseudoresistance, secondary hypertension, white-coat hypertension and medication non-adherence is an important step when diagnosing R-HTN. Most recently different phenotypes associated to R-HTN have been described, specifically refractory and controlled R-HTN and masked uncontrolled hypertension. Optimizing the three-drug regimen, including the diuretic treatment, adding a mineralocorticoid receptor antagonist as the fourth drug, a β-blocker as the fifth drug and an α1-blocker or a peripheral vasodilator as a final option when failing to achieve target blood pressure values are current recommendations regarding the correct management of R-HTN.

## 1. Introduction

Arterial hypertension (HTN), defined as office systolic blood pressure (BP) values ≥ 140 mmHg and/or diastolic BP values ≥ 90 mmHg, is known as the most important risk factor for cardiovascular (CV) diseases. Nowadays, the prevalence of HTN amounts to 1.13 billion people, or around 30–45% of the global population [1,2]. The World Health Organization concluded that HTN is the third cause of death worldwide (one in eight deaths being attributed to high BP) and this proportion is expected to increase over time [3]. Thus, the high prevalence of HTN and its subsequent complications are becoming a major problem for health systems across the world.

Resistant hypertension (R-HTN), a severe form of HTN, has been intensively studied since the early 1960s, when it was first defined. R-HTN is considered when failing to lower BP below 140/90 mmHg despite adequate lifestyle measures and optimal treatment with at least three antihypertensive medications of different classes, including a diuretic, typically a blocker of the renin-angiotensin system (angiotensin converting enzyme inhibitor or angiotensin receptor blocker) and a long-acting calcium channel blocker (CCB), at maximum or maximally tolerated daily doses administered at the appropriate dosing interval [4].

The 2018 European Society of Hypertension and European Society of Cardiology (ESH/ESC) Guidelines’ definition for R-HTN requires inadequate control of BP to be confirmed by ambulatory BP monitoring (ABPM) or home BP monitoring (HBPM) and adherence to therapy to be validated [4]. After excluding pseudoresistance and secondary forms of HTN, the prevalence of R-HTN is estimated to be less than 10% in hypertensive treated patients [4]. The increased prevalence during the last 30 years, despite the improvement of antihypertensive regimens, may be explained by the progressive ageing of the population and by the obesity pandemic.

## 2. Resistant Hypertension-Associated Phenotypes

**Figure 1:**
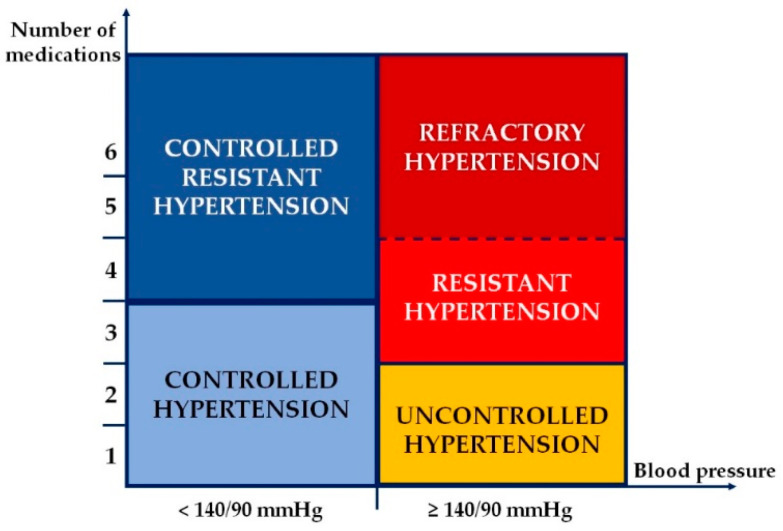
Hypertension classification based on blood pressure control and number of antihypertensive medications.

Different R-HTN-associated phenotypes have been described. The 2017 American College of Cardiology/American Heart Association (ACC/AHA) guideline introduced two new concepts: controlled R-HTN and refractory R-HTN [5]. Controlled R-HTN is considered in patients receiving at least four antihypertensive medications and achieving an adequate office BP control. Refractory R-HTN (rfR-HTN) refers to patients with elevated office BP values while on treatment with five or more antihypertensive drugs, including a long-acting thiazide-type diuretic, such as indapamide or chlorthalidone, or a mineralocorticoid receptor antagonist (MRA), such as spironolactone [5] (Figure 1). The prevalence of rfR-HTN has been estimated by a limited number of studies. The Spanish ABPM Registry database was initiated in 2004 and contained a total of 70,997 treated patients. A total of 11,972 patients fulfilled the standard criteria of R-HTN (16.9%) and 955 had rfR-HTN (7.9% of the total number of patients with R-HTN and 1.4% of the entire treated group) [6]. Similarly, the prevalence of rfR-HTN among the participants included in the REGARD (Reasons for Geographic And Racial Differences in Stroke) Study was 0.5% [7]. Compared with R-HTN, patients with rfR-HTN were younger, had a longer duration of HTN, a higher prevalence of obesity, diabetes mellitus, dyslipidemia, chronic kidney disease and target organ damage and previous history of CV events [6,7]. Thus, these patients require a greater medical attention among all patients with R-HTN.

Most recently, a new R-HTN-associated phenotype was described—masked uncontrolled HTN, referring to patients receiving four or more antihypertensive medications and achieving an adequate office BP control, but with elevated out-of-office BP [8]. Hence, screening of masked uncontrolled HTN should be performed in all patients with controlled R-HTN, due to higher CV risk and thus needing a more aggressive therapeutic strategy [9].

## 3. Pathophysiology of Resistant Hypertension

Various physiological mechanisms are involved in maintaining normal BP values; hence, their dysfunction may lead to the development of HTN. R-HTN is broadly attributed to two underlying processes: the renin-angiotensin-aldosterone system and sympathetic nervous system activation.

### 3.1. Role of the Renin-Angiotensin-Aldosterone System

Aldosterone is the most important mineralocorticoid hormone produced in the zona glomerulosa of the adrenal cortex as a response to angiotensin II, increased levels of extracellular potassium and corticotropin activity. Aldosterone regulates the sodium and potassium balance by binding and activating the mineralocorticoid receptor in the distal tubule and collecting duct of the kidney. Increased aldosterone production induces salt retention and, consequently, fluid retention, thus promoting elevated BP values. The detrimental actions of aldosterone include endothelial dysfunction, sympathetic activation and oxidative stress. In vascular smooth muscle cells, aldosterone displays several extrarenal effects by activating the expression of genes involved in inflammation, calcification and fibrosis [10,11,12]. Aldosterone is also involved in R-HTN due to several non-genomic proinflammatory effects that can maintain a pharmacologically resistant state [13].

### 3.2. Role of the Sympathetic Nervous System

Essential HTN is characterized by both impaired parasympathetic tone and increased sympathetic activation. Increased levels of the adrenergic neurotransmitters norepinephrine and epinephrine have also been found in normotensive patients with a family history of HTN [14]. The sympathetic system overdrive is mainly due to the positive chronotropic effects of norepinephrine. Adrenergic activation appears from early stages of HTN and becomes more visible as its severity increases by participating in the development of target organ damage [15]. Potential mechanisms responsible for the sympathetic activation in HTN are: cardiopulmonary reflex dysfunction, chemoreceptor stimulation, baroreflex dysfunction, central factors, insulin and leptin [15]. Studies also showed a statistically significant activation of renal sympathetic system in patients with R-HTN when using norepinephrine regional spillover techniques [16].

Activation of the adrenergic nervous system is involved in the development of vascular remodeling, endothelial dysfunction and arterial stiffness in hypertensive state [17]. For a similar BP elevation, sympathetic activation is more pronounced in patients with R-HTN compared to those with controlled HTN [18].

### 3.3. Role of Genetics

In recent years, new methodologies have been developed making it possible to employ genome-wide association studies (GWAS) for testing the correlation between thousands of common gene variants and common characteristics in samples from different populations. GWAS assume genetic expression and epigenetic analysis in different tissues, and this way, thousands of genes and pathways with potential biological relevance may be discovered. However, the results for BP phenotypes are scarce as the gene variants explain only 2.2% of BP variation and are, thus, not applicable for clinical implementation [19]. Moreover, given the fact that R-HTN is a special phenotype, it is probably caused by both common and rare gene variants. While common gene variants may be identified by GWAS, the rare ones may not. Therefore, improving this method in order to identify new gene variants and understand the relation between them and antihypertensive medication is strongly needed. Furthermore, although it is difficult to translate pharmacogenomics findings on R-HTN genotypes into clinical practice, this will probably be a very important step in the management of this pathology as it may improve the BP response to drugs and CV outcomes.

## 4. Diagnostic Approach of Resistant Hypertension

**Figure 2:**
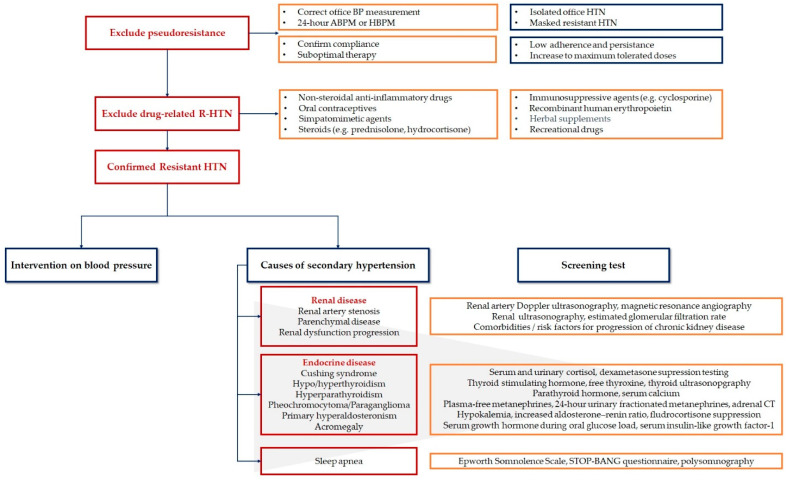
Diagnostic approach of resistant hypertension. ABPM: ambulatory blood pressure monitoring; CT: computed tomography; HBPM: home blood pressure monitoring; HTN: hypertension; R-HTN: resistant hypertension. Created based on information from literature [4,20,21].

The classification of R-HTN has become much easier after the new definition of R-HTN was published in the 2018 ESH/ECS guideline [4]. When assessing a patient with possible R-HTN, we must consider many important steps to define the diagnostic approach (Figure 2).

### 4.1. Confirm Treatment Resistance

According to the definition, elevated values of office BP are not sufficient for establishing the diagnosis of R-HTN, and the inadequate control of BP needs to be confirmed by ABPM and, if not available, HBPM in patients whose adherence to therapy has been confirmed [4]. These types of measurements can lead to at least three situations:

- White-coat uncontrolled HTN—in patients with office BP above goal, but out-of-office BP measured by ABPM or HBPM are below target values;
- Masked uncontrolled HTN—in patients with adequate office BP control, but with elevated out-of-office BP measured by either ABPM or HBPM;
- Sustained uncontrolled HTN—in patients whose BP values are elevated at both office and out-of-office measurements.

Another HTN classification based on BP control and number of antihypertensive medications is the following (as shown in both Figure 1 and Figure 2) [4,5]:

- Uncontrolled R-HTN—in patients with office BP ≥ 140/90 mmHg and/or 24-h ambulatory BP ≥ 130/80 mmHg, taking three or more antihypertensive medications of different classes, including a diuretic, and usually a long-acting calcium channel blocker and a blocker of the renin-angiotensin system blocker at maximal or maximally tolerated doses;
- Controlled R-HTN—in patients with office BP < 140/90 mmHg and/or 24-h ambulatory BP < 130/80 mmHg, taking at least four antihypertensive medications of different classes.

### 4.2. Exclude Pseudoresistance

The main triggers for pseudoresistance are poor office BP measurement technique, the “white-coat” effect, marked brachial artery calcification, clinical inertia, or suboptimal adherence or non-adherence to lifestyle measures or antihypertensive treatment.

Common errors in office BP measurement technique are: not allowing the patient to rest for at least five minutes prior to measurement, poor sitting position with unsupported back, full bladder, measuring BP with the arm unsupported at heart level, using cuffs that are too small for the arm circumference, placing the cuff over clothing or engaging the patient in conversation during BP measurement. Another cause of pseudoresistance is marked brachial artery calcification, especially in the elderly with heavily calcified arteries, which leads to peripheral arterial incompressibility and elevated BP values at conventional BP measurement.

Before the diagnosis of R-HTN is confirmed, adherence to treatment must be evaluated. Studies show that 31 to 50% of hypertensive patients have either partial adherence or complete non-adherence to lifestyle measures or antihypertensive treatment [22]. This situation can be a consequence of multiple factors: demographic factors (including age, gender, education), condition-related (HTN, at least in early stages, is an asymptomatic disease; long-term drug administration for many chronic illnesses), therapy-related (complexity of drug regimen—number of drugs, dosing, adverse side effects), cost barriers, inadequate patient–clinician relation and clinician inertia (failure to intensify treatment when needed resulting in submaximal doses or inadequate treatment regimen) [23]. Therefore, non-adherence is an indicator of poor prognosis and correlates with CV outcomes, leading to repeated hospital admissions [24]. Adherence should be associated with persistence when administrating the drug regimen in order to optimize prognostic benefits. Adherence to treatment can be measured by both indirect and direct methods. Indirect methods refer to self-reported questionnaires such as the Moriski Medication Adherence Questionnaire or the Hill-Bone Compliance Scale, or an honest discussion between patient and clinician with monitoring of prescription refills and pill count [25]. Direct methods for evaluating adherence refer to administration of treatment under strict supervision, using a Medication Event Monitoring System, or by monitoring the plasma or urine concentrations of drugs. The Medication Event Monitoring System uses various sensors which are placed in the medication and record the date, the hour and the number of times the bottle has been opened, although this does not guarantee the actual ingestion of medications [26]. When possible, biochemical monitoring of drugs or their metabolites in plasma or urine can be performed (e.g., urine fluorometry, high-performance liquid chromatography and mass spectrometry urine analysis), even if these methods determine only if the drug is present or absent, not the actual persistence in treatment. There are several methods for improving adherence: using combination pills that preferably require only one daily administration, or using low-cost or generic antihypertensive medications, especially in patients with multiple chronic illnesses.

### 4.3. Exclude Drug-Related Resistant Hypertension

Several classes of medications can increase BP and, thus, lead to or aggravate R-HTN. These types of medications include prescription drugs (oral contraceptives, sympathomimetic agents, nonsteroidal anti-inflammatory drugs, steroids, immunosuppressive agents, recombinant human erythropoietin, etc.) and non-prescription drugs (herbal supplements, recreational drugs, etc.) [21]. However, the BP response to these medications can be highly individualized. While the majority of people show little to no effect when taking these types of drugs, others show severely increased BP values which can cause or worsen R-HTN.

### 4.4. Exclude Secondary Causes of Hypertension

After excluding pseudoresistance and drug-related HTN, screening for secondary causes of HTN is essential. Figure 2 illustrates the most frequent causes of secondary HTN as well as the screening methods for each pathology.

Screening for this type of HTN should be guided by clinical findings as, in most situations, curative treatment of secondary HTN does not imply the normalization of BP values, especially if the disease had evolved for a long period of time and, thus, led to possible vascular or organ damage.

## 5. Therapeutic Approach of Resistant Hypertension

Once a patient was diagnosed with R-HTN, a set of nonpharmacological, pharmacological and, if needed, device-based strategies can be applied in order to obtain optimal control of BP.

### 5.1. Nonpharmacological Strategies

Nonpharmacological measures are commonly recommended as they are the cornerstone for both preventing and treating HTN. We will discuss below several lifestyle factors that influence BP values.

#### 5.1.1. Weight Reduction

Obesity is, undeniably, one of the most evident and yet neglected problems of public health. The number of obese adults was estimated at 500 million in 2016, with an alarming increasing trend among children. It is estimated that by 2030, the epidemic of “globesity” will amount to approximately 1.5 billion people [27].

Even if the pathophysiological mechanisms that link obesity to BP are complex, the key element might be the distribution of body fat. Vague was the first to raise this issue back in 1956, when finding the correlation between metabolic and CV complications and the phenotype of obesity—android or gynoid [28]. Since then, the waist-to-hip ratio and afterwards the waist circumference were more frequently used. Central obesity (also known as visceral obesity) is defined as waist circumference ≥ 94 cm in men and ≥ 80 cm in non-pregnant women.

Studies also demonstrated that there is an almost linear correlation between body mass index and BP values. With every 1.7 kg/m^2^ and 4.5 cm waist circumference in men and 1.3 kg/m^2^ and 2.5 cm in waist circumference in women, the systolic BP increases with 1 mmHg [29].

The National Health and Nutrition Examination Survey (NHANES) and the Spanish ABPM Registry demonstrated that a body mass index above 30 kg/m^2^ doubles the risk of true R-HTN [30]. Hence, weight reduction is advisable for all patients, ideally achieving a body mass index below 25 kg/m^2^. Even if the BP-lowering effect appears in early stages of weight loss, the long-term effects being more attenuated, persevering in weight loss is extremely important [31]. However, studies providing the effectiveness of weight loss in R-HTN are not yet available.

#### 5.1.2. Dietary Sodium Restriction

One of the most important measures in reducing BP values is, undeniably, sodium restriction, given the fact that hydro-saline retention is involved in the pathophysiology of HTN. The 2018 ESH/ECS Guidelines recommend a daily sodium intake limited to 5 g per day [4]. In contrast, the 2017 ACC/AHA Guidelines advises that the optimal goal is a sodium intake less than 1.5 g per day, but aims for at least 1 g per day reduction in most hypertensive adults, hence obtaining a BP reduction of 5–6 mmHg, or a similar reduction to that provided by antihypertensive drug monotherapy [5].

#### 5.1.3. The DASH Diet

The Dietary Approaches to Stop Hypertension (DASH) diet was designed as a 11-week program in 459 individuals with mild and untreated HTN who were randomly assigned to one of three types of diet. The first one—the DASH diet—was based on fruits, vegetables and low-fat dairy products with reduced saturated and total fat, the second was based on fruits and vegetables rich in potassium and fiber, while the third one was based on a typical American diet (the control diet). Each diet accounted a total sodium intake of about 3 g per day. The DASH diet lowered BP by 6/3 mmHg, while the fruits and vegetables diet lowered BP by 3/1 mmHg [32]. In addition, the DASH diet also showed efficiency in weight loss [33].

#### 5.1.4. Physical Activity

Physical activity has been consistently studied and shown to reduce both systolic and diastolic BP, being beneficial for preventing and treating HTN as well as lowering CV risk and mortality. There are multiple types of physical activities, such as dynamic or aerobic exercises (walking, running, swimming, cycling, etc.) and isometric or resistance exercises (weight lifting, body building, etc.), and each of these showed BP-lowering properties. When hypertensive patients perform 90 to 150 min of aerobic exercise per week, they can register a reduction of BP values by 5–8 mmHg, whereas when performing an isometric resistance exercise over the course of 8–10 weeks, the systolic BP can be reduced by 4–5 mmHg [34]. The benefits of physical activity in R-HTN are scarce, but the few studies that have been conducted showed promising results [35].

#### 5.1.5. Other Risk Factors

Other lifestyle measures that are found to be helpful in reducing BP values are: limiting alcohol consumption to less than 14 units per week in men and less than 8 units per week in women, avoiding binge drinking, avoiding excessive caffeine, smoking cessation and having a healthy sleep pattern with at least six hours of sleep per night [4,5].

#### 5.1.6. Psychological Counseling

Beyond lifestyle measures, the psychosocial factors play an important role in treating HTN, mainly because they may influence adherence and/or persistence to therapy. Psychosocial stress, anger, anxiety and especially depression are common in adults and are frequently associated with the development of HTN, hence affecting the individual health and the quality of life [36]. A meta-analysis showed that depression increases the risk of HTN with 42% [37]. Other studies show that both depression and poor adherence to treatment double the risk of stroke, whereas omission of one or more antihypertensive drugs due to depression may lead to poor BP control and thus increase CV risk [38]. In patients with multidrug regimens, it is essential to evaluate their lifestyle, psychological well-being, possible afflictions such as depression or anxiety and compliance to therapy before defining R-HTN. Therefore, psychological counseling should not be neglected and must be taken into consideration when treating hypertensive patients.

### 5.2. Pharmacological Strategies

**Figure 3:**
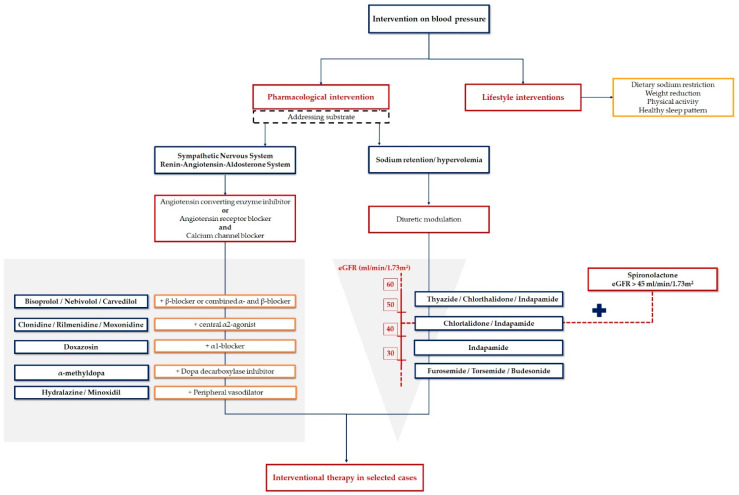
Therapeutic approach of resistant hypertension. eGFR: estimated glomerular filtration rate. Created based on information from literature [4,21].

All hypertensive patients require drug therapy in addition to lifestyle measures to achieve an adequate BP control. We will discuss below the pharmacological strategies that should be applied in a stepwise manner in order to achieve therapeutic success in R-HTN (Figure 3).

#### 5.2.1. Optimizing the Three-Drug Regimen

When treating R-HTN assuring that the diagnostic criteria have been fulfilled alongside with optimal nonpharmacological measures is essential. Therefore, the treatment of R-HTN includes at least three antihypertensive medication of different classes, including a diuretic, and usually a blocker of the renin-angiotensin system and a CCB, at maximum or maximally tolerated daily doses and at the appropriate dosing interval. Note that when HTN is associated with atrial fibrillation or heart failure, the initial triple therapy may be different. Additionally, adherence to treatment must be improved (e.g., a combination, if possible, of all medications in a single pill) [39].

#### 5.2.2. Optimizing the Diuretic Treatment

Thiazide-like diuretics (chlorthalidone, indapamide) are considered the first-line diuretics in R-HTN having a longer half-life when compared to thiazide diuretics (hydrochlorothiazide), which translates into a better control of BP [40].

When choosing the appropriate diuretic, one must take into account the estimated glomerular filtration rate (eGFR). In patients with eGFR higher than 30 mL/min/1.73 m^2^, it is advisable to use thiazide or thiazide-like diuretics, whereas in patients with eGFR below 30 mL/min/1.73 m^2^, loop diuretics (furosemide, bumetanide or torsemide) are preferred. Even though hydrochlorothiazide is the most prescribed thiazide diuretic, switching to chlorthalidone might be a better choice due to its longer acting effect (also having an effect on nocturnal BP), its higher efficiency and superiority in preventing CV events and left ventricle hypertrophy [41,42]. Electrolytes should be monitored regularly as both thiazide and thiazide-like diuretics can induce hypokalemia. Additionally, among all loop diuretics, torsemide might be the best option given its longer duration of action, once daily dosing, and a more reliable bioavailability when compared to others [43].

#### 5.2.3. Adding a Mineralocorticoid Receptor Antagonist

When target BP values cannot be obtained with the three-drug regimen, a fourth medication needs to be added. Therefore, given the fact that mineralocorticoid receptor blockade is extremely important in hypertensive patients, mineralocorticoid receptor antagonists are one of the front-runner medication classes for treating R-HTN [4,5].

The PATHWAY-2 study (Prevention and Treatment of Hypertension with Algorithm Based Therapy) included patients with uncontrolled R-HTN taking optimal treatment with at least three antihypertensive medication of different classes who were randomized to a double-blinded, four-way cross-over comparison of 3 months each of placebo, spironolactone (25 or 50 mg), bisoprolol (5 or 10 mg) and doxazosin modified release (4–8 mg) [44]. Spironolactone was superior to the other two classes of agents and to placebo in patients with uncontrolled R-HTN, and reduced home systolic BP by 8.70 mmHg more than placebo, 4.48 mmHg more than bisoprolol and 4.03 mmHg more than doxazosin [44]. PATHWAY-2 also demonstrated that titration up to 50 mg, if tolerated, is clinically appropriate for patients who remain uncontrolled with the lower dose [44].

Even though spironolactone is very useful in treating R-HTN, it has certain limitations and contraindications. It should not be used in patients with eGFR lower than 45 mL/min/1.73 m^2^, as it may lead to deterioration of renal function and hyperkalemia. Studies show that potassium binders such as patiromer are extremely efficient in patients with R-HTN and chronic kidney disease by enabling patients to continue the treatment with spironolactone with a lower risk of hyperkalemia [45]. Additionally, note that when using spironolactone, several side effects may occur such as gynecomastia and erectile dysfunction in men and menstrual irregularities in women.

PATHWAY-2 included several sub-studies aimed to explore the underlying causes of R-HTN, including the role of aldosterone and excess fluid retention as important mediator of treatment resistance. The first sub-study demonstrated the relation between the baseline aldosterone-renin ratio and baseline renin level and the BP response to spironolactone [46]. The second sub-study showed that R-HTN is mainly attributable to excess fluid retention due to increased levels of aldosterone, although prior studies showed that a diet low in sodium and high in potassium reduced BP despite aldosterone excess [46]. The third sub-study evaluated the antihypertensive effect of amiloride, another mineralocorticoid receptor antagonist. Amiloride-sensitive sodium channels are present in both endothelial and vascular smooth muscle cells and display various mechanisms through which mineralocorticoid receptor activation can affect vascular function and consequently BP [47]. The patients received 10 to 20 mg amiloride (only 146 of the patients included in the study) and had reduction of home systolic BP of 20.4 mmHg, similar with the 18.3 mmHg reduction secondary to spironolactone. Hence, amiloride can be as effective as spironolactone as a fourth medication in treating R-HTN and can be used as an alternative to spironolactone, if the latter is not well tolerated. Additionally, small studies concluded that eplerenone is effective in treating R-HTN and may be considered when adverse effects are noted with spironolactone treatment [48]. Because eplerenone has a shorter life compared to spironolactone, it should be administered twice a day for an optimal effect.

#### 5.2.4. Adding a β-Blocker or a Combined α- and β-Blocker

The fourth therapeutic option consists of adding a cardio-selective β-blocker or a combined α- and β-blocker, especially if the basal heart frequency is above 70 beats per minute, and in case they are not already part of the treatment for a different pathology such as heart failure with reduced ejection fraction, atrial fibrillation, or coronary artery disease. The 2018 ESH/ECS guideline favors the use of bisoprolol as it was shown in the PATHWAY-2 study [4,46]. However, it may be more reasonable to add a β-blocker with an enhanced vasodilator effect, such as nebivolol or a combined α- and β-blocker, such as carvedilol.

In case β-blockers are ineffective, contraindicated or not tolerated, central α-2 agonists such as clonidine, rilmenidine or moxonidine can be considered. A patch form of clonidine is preferred because of a lower risk of rebound after discontinuation or non-adherence [49]. More recently, the Resistant Hypertension Optimal Treatment (ReHOT) study compared spironolactone and clonidine as the fourth drug in R-HTN and showed that they lower office and ambulatory BP in a similar way, although spironolactone achieves greater decrease in both systolic and diastolic 24-h BP as well as in diastolic daytime BP, but does not register a night-time difference [50].

Rarely, if there is a specific contraindication to β-blockers, a non-dihydropyridine calcium channel blocker can be added [51].

#### 5.2.5. Adding an α1-Blocker or a Peripheral Vasodilator

The fifth therapeutic option consists of adding a new therapeutic class with a more potent antihypertensive effect, but also with greater side effects.

The first option would be α1-blockers such as doxazosin. The starting dose for doxazosin is 1 mg per day, preferably in the evening in order to minimize the risk of orthostatic hypotension and/or syncope, especially in older adults. The dose can increase every one or two weeks until a maximum dose of 16 mg per day, although many patients only need 4 to 8 mg per day in order to obtain adequate control of BP. The α1-blockers may be considered in patients with concomitant benign prostatic hyperplasia [4].

α-methyldopa is frequently used in pregnancy HTN, but it can also be useful in R-HTN. The starting dose is 250 mg, once daily, preferably in the evening for one or two weeks to minimizing the effect of sedation and to monitor tolerance. Then, the doses can be slowly increased until a maximum dose of 2 g per day [52].

If BP is still uncontrolled with the aforementioned medications, hydralazine can be another option. Doses less than 150 mg per day decrease the risk of developing drug-induced lupus-like syndromes. Another major disadvantage of hydralazine is that the direct arterial vasodilation determines the activation of the sympathetic nervous system with subsequent tachycardia and hydro-saline retention [52]. Thus, hydralazine should always be associated with an appropriate diuretic and a β-blocker. The addition of nitrates to hydralazine in cases of heart failure with reduced ejection fraction is generally recommended [52]. In case BP is still uncontrolled on a combination of maximal or maximally tolerated doses of thiazide-like diuretic, a blocker of the renin-angiotensin system, a long-acting calcium channel blocker, spironolactone, and a β-blocker and/or centrally acting α-agonist, switching to another peripheral vasodilator, minoxidil, comes as the last option [5]. Similarly to hydralazine, minoxidil causes the activation of the sympathetic nervous system and sodium retention and must be associated with both β-blockers and loop diuretics [53]. The major side effects of minoxidil are hirsutism and the necessity of frequent dosing, which may lead to non-adherence and discontinuation of treatment.

### 5.3. Device-Based Therapy

Since the activation of the sympathetic nervous system is one of the key mechanisms for the development and progression for both essential HTN and R-HTN, there has been an emerging need to create a series of device-based therapies to disrupt this mechanism and, thus, control BP.

#### 5.3.1. Renal Sympathetic Denervation

The purpose of renal sympathetic denervation is to ablate the sympathetic nerves that are localized close to the renal arteries by radiofrequency or ultrasound waves emitted by an endovascular catheter and, therefore, interrupt the sympathetic activation. This therapy was evaluated in patients with R-HTN taking 4–5 antihypertensive drugs by three studies called SYMPLICITY (Catheter-based renal sympathetic denervation for resistant hypertension: a multicenter safety and proof-of-principle cohort study). It was first used in a human feasibility trial (SYMPLICITY HTN-1) and then in a larger randomized prospective study (SYMPLICITY HTN-2) and showed a reduction in office BP and a good safety profile when using this device [54,55]. However, these studies were uncontrolled and lacked validation by ABPM. The first sham-controlled randomized clinical trial (SYMPLICITY HTN-3) showed little to no effect on BP in patients with severe R-HTN [56]. Profound analyses have been carried out to explain the contradictory results. Possible explanations are incomplete nerve ablation due to poorly designed catheter electrodes or inexperienced operators, non-adherence to the multidrug pharmacological regimen and not including ABPM in the efficacy analysis.

Since then, the Renal Denervation for Hypertension (DENER-HTN) Trial showed a statistically significant reduction in daytime ambulatory systolic BP (–5.9 mmHg) in hypertensive patients on a three-drug regimen randomized to renal sympathetic denervation, when compared to patients taking four antihypertensive medications without renal denervation [57].

Recently, catheters have been re-designed to allow complete circumferential renal nerve ablation. SPYRAL-OFF MED Pivotal trial is a prospective, sham-controlled, randomized trial that uses this type of catheter and evaluates renal sympathetic denervation independently of antihypertensive drugs. This trial showed significant BP reduction (–4.7 mmHg in 24-h ambulatory BP and –6.6 mmHg in office BP) after 3 months of follow-up [58]. Although these results are promising, further studies need to be carried out in patients with R-HTN that are followed up longer.

Radiofrequency from renal sympathetic denervation can be replaced with ultrasound, as new research demonstrated—Paradise Renal Denervation System, ReCor Medical. This technique also showed a reduction of BP in its own proof-of-concept trial [59].

#### 5.3.2. Carotid Baroreceptor Activation Therapy

The carotid baroreceptor activation reflex is triggered by a rapid pressure within the carotid body, which leads to a reduction of sympathetic signals to the vessels, heart and kidneys, and hence, to the reduction of BP. There are two types of devices that evaluate baroreceptor activation therapy: an implantable pulse generator (Rheos and BAROSTIM NEO systems) and an endovascular device (MobiusHD).

The Rheos system consists of a battery-powered implantable pulse generator that electrically activates the carotid baroreflex. Although the Rheos Pivotal Trial showed no superiority of baroreceptor activation therapy for reducing BP compared to medical therapy after a six-months follow-up, more than half achieved a systolic BP less than 140 mmHg [60].

#### 5.3.3. Central Arteriovenous Anastomosis

The central arteriovenous anastomosis assumes unloading the arterial vascular bed into the venous system. The ROX Coupler utilizes a stent-like nitinol device that is placed through the anastomosis that has been created between the femoral artery and vein and demonstrated efficacy in lowering BP [61]. However, there is a need for a longer follow-up given the risk of right heart failure.

## 6. Looking into the Future

The identification of new molecules to lower BP and CV risk is a continuous concern of the pharmaceutical industry. Notwithstanding a large spectrum of new therapeutic agents used in HTN, it seems difficult to create efficient and competitive molecules that go beyond the clinical phases of studies.

Several studies evaluated the antihypertensive effect of the association between angiotensin-converting enzyme inhibitors and neutral endopeptidase inhibitors. The first class of drugs inhibit the angiotensin-converting enzyme leading to a decrease of angiotensin-II, thus improving vasoconstriction and reducing sodium retention. The second class of drugs prevents the degradation of natriuretic peptides into metabolites and promotes vasodilation, increases sodium excretion and displays antihypertrophic and antifibrotic properties. Their effect on BP is synergic and the protection of organ damage is superior. The biggest problem when combining these two classes is the growing number of patients with angioedema because both angiotensin-converting enzyme inhibitors and the neutral endopeptidase inhibitors are involved in the degradation of bradykinin. The Omapatrilat Cardiovascular Treatment vs. Enalapril (OCTAVE) trial compared the antihypertensive effect of omapatrilat versus enalapril and although the antihypertensive effect was superior, angioedema appeared more frequently with the first (2.17% versus 0.68%) [62]. These observations led to a new association between angiotensin-II receptor blockers and neprilysin inhibitors. The most studied molecule is sacubitril/valsartan, which was shown to provide greater benefits in heart failure with reduced ejection fraction, but also promising results in HTN [63]. The PARAMETER (Prospective Comparison of Angiotensin Receptor Neprilysin Inhibitor With Angiotensin Receptor Blocker Measuring Arterial Stiffness in the Elderly) study demonstrated superiority of sacubitril/valsartan versus olmesartan in reducing office and ambulatory central aortic and brachial pressures in elderly patients with systolic HTN and arterial stiffness. A recent study found that sacubitril/valsartan is safe and well tolerated and showed that its effect on BP is dose dependent [64]. Sacubitril/valsartan is still under research for a larger category of patients with severe HTN, sleep apnea, post-myocardial infarction or peripheral artery disease.

As discussed before, the mineralocorticoid receptor antagonists have proved their efficiency in controlling BP. At this moment, among the novel nonsteroidal mineralocorticoid receptor antagonists, dihydronaphthyridine finerenone (BAY94-8662) has accumulated the most evidence. Finerenone is distributed in both cardiac and renal tissues, and it was initially developed for the treatment of heart failure with reduced ejection fraction and mild to moderate chronic kidney disease [65]. Finerenone reduces the expression of pro-inflammatory and pro-fibrotic markers and decreases proteinuria, including at doses that do not significantly influence BP [66]. Despite the favorable effects on cardiac hypertrophy or fibrosis and the reduction of B-type natriuretic peptide and N-terminal pro B-type natriuretic peptide levels, finerenone has a minimal effect on BP. Studies show that finerenone effect on systolic BP is similar to placebo, while spironolactone significantly reduces office systolic BP [65]. When needed, finerenone can be used as an alternative to steroidal mineralocorticoid receptor antagonists as it displays a lower incidence of hyperkalemia [10].

A functional renin-angiotensin system exists inside the brain and controls the cardiac functions and the body fluid homeostasis [67]. Aminopeptidase A, a membrane-bound zinc metalloprotease, is involved in the conversion of angiotensin-II to angiotensin-III. Hyperactivity of the brain renin-angiotensin system and consequently of the brain aminopeptidase A led to the development and progression of HTN in various experimental animal models [67]. A specific and selective aminopeptidase A inhibitor called EC33 does not cross the blood–brain barrier and inhibits aminopeptidase A enzymes only after intracerebroventricular injection. Hence, an active prodrug of EC33, called firibastat, was developed [67]. When administered in animal models, firibastat crosses the blood–brain barrier and generates two active molecules of EC33 which inhibit the brain aminopeptidase A activity and block the formation of brain angiotensin-III [68]. Firibastat decreases BP in both spontaneously hypertensive rats and in deoxycorticosterone acetate salt hypertensive rats, but does not lower BP in normotensive rats [69,70]. Clinical phase II trials showed the efficacy of firibastat for lowering BP in hypertensive patients. A total of 256 overweight or obese patients were included in a phase II study and received firibastat for eight weeks. For the first two weeks, the patients were treated only with firibastat (250 mg twice per day); the next two weeks the dose was doubled if automated office BP was higher than 140/90 mmHg. After another month, 25 mg of hydrochlorothiazide was added if automated office BP was higher than 160/110 mmHg. After 8 weeks, firibastat lowered automated office BP by 9.5/4.2 mmHg in 85% of the patients treated with firibastat alone [71]. The main limitation of this study was the absence of a control group. Phase III trials need to be conducted as the inhibition of aminopeptidase A can represent a new direction for treating R-HTN.

## 7. Conclusions

Patients with R-HTN present a higher mortality rate compared to non-R-HTN patients due to increased CV risk and its associated adverse outcomes—greater risk of developing chronic kidney disease, heart failure, stroke or myocardial infarction. Excluding pseudoresistance, secondary and white-coat hypertension is crucial when diagnosing R-HTN. Ensuring adherence to lifestyle measures and drug regimen is an important factor in achieving adequate BP control and avoiding unnecessary intensification of treatment. Optimizing the three-drug regimen, switching to a more potent diuretic, adding a mineralocorticoid receptor antagonist as the fourth drug, a β-blocker or combined α- and β-blocker as the fifth drug and an α1-blocker or a peripheral vasodilator as a final option when failing to achieve target BP values, are current recommendations that regard the correct management of R-HTN. Until the approval of new medications, we must optimize the usage of the existing ones by combining them more rationally and in optimal doses, in order to improve adherence to treatment and, thus, control BP. Device-based therapies such as renal sympathetic denervation and carotid baroreceptor activation therapy show promising results but need further studies to confirm their efficacy and safety in clinical practice.

## Data Availability

Not applicable.

## Abbreviations

ABPM—ambulatory blood pressure monitoring; ACC/AHA—American College of Cardiology/American Heart Association; BP—blood pressure; CCB—long-acting calcium channel blocker; CV—cardiovascular; DASH—Dietary Approaches to Stop Hypertension; eGFR—estimated glomerular filtration rate; ESH/ESC—European Society of Hypertension and European Society of Cardiology; GWAS—genome-wide association studies; HBPM—home blood pressure monitoring; HTN—arterial hypertension; MRA—mineralocorticoid receptor antagonist; PATHWAY–2—Prevention and Treatment of Hypertension with Algorithm Based Therapy; rfR–HTN—refractory resistant hypertension; R–HTN—resistant hypertension; SYMPLICITY—Catheter-based renal sympathetic denervation for resistant hypertension: a multicentre safety and proof-of-principle cohort study.
